# Efficient and stable tin perovskite solar cells enabled by amorphous-polycrystalline structure

**DOI:** 10.1038/s41467-020-16561-6

**Published:** 2020-05-29

**Authors:** Xiao Liu, Yanbo Wang, Tianhao Wu, Xin He, Xiangyue Meng, Julien Barbaud, Han Chen, Hiroshi Segawa, Xudong Yang, Liyuan Han

**Affiliations:** 1 State Key Laboratory of Metal Matrix Composites, Shanghai Jiao Tong University, 800 Dong Chuan Road, Shanghai, 200240 China; 2 Photovoltaic Materials Group, Center for Green Research on Energy and Environmental Materials, National Institute for Materials Science, Tsukuba, Ibaraki 305-0047 Japan; 3 Research Center for Advanced Science and Technology, University of Tokyo, Tokyo, 153-8904 Japan; 4 School of Materials Science and Engineering, Henan Institute of Advanced Technology, Zhengzhou University, Zhengzhou, 450001 China; 5 Faculty of Pure and Applied Science, University of Tsukuba, Tsukuba Ibaraki, 305-8571 Japan

**Keywords:** Chemistry, Materials science, Nanoscience and technology, Optics and photonics, Physics

## Abstract

Tin perovskite solar cells (TPSCs) have triggered intensive research as a promising candidate for lead-free perovskite solar cells. However, it is still challenging to obtain efficient and stable TPSCs because of the low defects formation energy and the oxidation of bivalent tin; Here, we report a TPSC with a stable amorphous-polycrystalline structure, which is composed of a tin triple-halide amorphous layer and cesium-formamidinium tin iodide polycrystals. This structure effectively blocks the outside oxygen, moisture and also suppresses the ion diffusion inside the devices. In addition, its energy level benefits the charge extraction and transport in TPSCs. This design enabled us to obtain the certified quasi-steady-state efficiency over 10% for TPSCs from an accredited certification institute. The cell was stable, maintaining 95% of the initial PCE after operation at the maximum power point under AM 1.5 G simulated solar light (100 mWcm^−2^) for 1000 hours.

## Abstract (web summary)

Tin based perovskite solar cells could be promising replacements for lead based counterparts due to their lower toxicity, but their efficiencies are much lower. Here, Liu et al. report stable tin based perovskite solar cells with efficiency over 10% by designing amorphous-polycrystalline nanostructures.

## Introduction

Organic–inorganic lead halide perovskite solar cells (PSCs) have achieved great progress in the conversion of solar light into electricity at low-cost^1–6^. This photovoltaic technology still suffers from the potential danger of lead toxicity, which casts a shadow on its future industrialization^7,8^. Recently, environmental friendly tin perovskite solar cells (TPSCs) have emerged as a promising candidate for the lead-free PSCs^9–15^. However, it is difficult to obtain efficient and stable TPSCs because of the low defects formation energy and oxidation from Sn^2+^ to Sn^4+^ during the crystallization of tin perovskites^16,17^.

Efforts have been made to enhance the efficiency and stability, such as the application of 2D–3D hierarchy structure^18–20^, antioxidants^21–24^, and reducing agents^25,26^. In addition, mixed-cation engineering has been proved to stabilize the perovskite phase^27,28^. Currently, the authoritative stabilized certification of the TPSCs performance is still missing, since the cells sent to accredited institutes are required to reach a steady output and undergo repeated current–voltage (*I–V*) tests until the values collected under operational conditions are stable over time. The lack of stabilized certified results leaves an open question on deviations in evaluation of TPSCs. Therefore, it is highly desired to overcome the limitation on the efficiency and stability for the future application of TPSCs.

Here, we fabricate efficient and stable TPSC based on an amorphous-polycrystalline structure composed of a tin triple-halide amorphous layer and CsFASnI_3_ polycrystals. This structure demonstrates its blocking effect on the outside moisture, oxygen, and the ion diffusion inside the devices. As a result, we achieve the certified quasi-steady-state efficiency over 10% (10.08%; Newport Laboratory, USA) for TPSCs, which retained over 95% of its initial PCE after working at the maximum power point under simulated sunlight of AM 1.5 G (100 mW cm^−2^) for 1000 h.

## Results

### Crystallization of tin perovskites

**Fig. 1:**
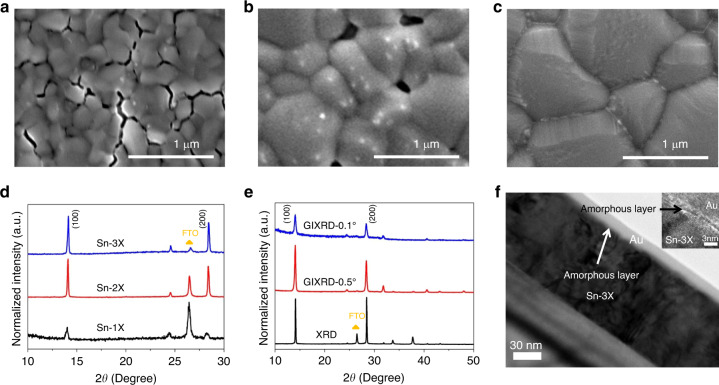
Crystallization of tin perovskite films. **a** SEM images of Sn-1X, **b** Sn-2X, and **c** Sn-3X. **d** The XRD patterns of the corresponding three samples. **e** The XRD and GIXRD patterns of Sn-3X; the incident angles of GIXRD are 0.5° and 0.1°, respectively. **f** TEM and HRTEM (inset) images of Sn-3X film sample fabricated by focused ion beam technique.

Three tin perovskite films were fabricated and denoted as Sn-1X (CsFASnI_3_; with I^−^ only), Sn-2X (CsFASnI_3_—10 mol% SnF_2_; F^−^ and I^−^), and Sn-3X (CsFASnI_3_—10 mol% SnF_2_-20 mol% SnCl_2_; F^−^, Cl^−^, and I^−^), respectively. One thing that needed to be claimed is that EDAI_2_ was added to all the samples (Sn-1X, Sn-2X, and Sn-3X) with the same molar ratio of 1%. It was found that EDAI_2_ has the effects of curing pinhole and passivating surface defects in tin perovskites, as pointed at previous work^13^. In this case, we usually use EDAI_2_ as an additive during the fabrication of tin perovskite films. Since all the samples here were added EDAI_2_ with the same concentration, we could exclude the effects of EDAI_2_ on the differences between Sn-1X, Sn-2X, and Sn-3X, as are shown below. Figure 1a–c show the scanning electron microscopy (SEM) images of the three samples. Obviously, Sn-1X film showed poor crystallization with large gaps between grains (Fig. 1a), which can notoriously deteriorate the separation and collection of photo-generated charge carriers. In contrast, the morphology of Sn-2X film was improved but pin-holes appeared with white dots on the surface (Fig. 1b), which could be ascribed to the phase segregation induced by SnF_2_ according to previous reports^29,30^. When the amount of SnF_2_ was further increased to 20 mol% in Sn-2X film, the number of pin-holes reduced but more white dots were observed (Supplementary Fig. 1a). The white dots would increase the surface roughness and the pin-holes can lead to the deterioration of TPSCs. For the Sn-3X perovskite film, a pin-hole free and dense layer with a large grain size of 600–1000 nm was obtained (Fig. 1c). We also fabricated Sn-3X films with different amount of SnCl_2_. Pin-holes still remained in the sample of Sn-3X film with 10 mol% SnCl_2_ while for the Sn-3X film with 30 mol% SnCl_2_, non-uniform thick-layered structure could be observed (Supplementary Fig. 1b, c). The corresponding XRD patterns for these Sn-3X films were also provided in Supplementary Fig. 2. To verify whether it will also work without SnF_2_, we fabricated the tin perovskites using only SnCl_2_ as the additive. However, the crystallization was found to be poor with lots of pin-holes (Supplementary Fig. 1d–f). One thing that concerned us is that, different from the morphology of Sn-2X film, layered structure seemed to be formed on the surface of Sn-3X film, which will be discussed in the next section.

The X-ray diffraction (XRD) pattern indicates that Sn-1X, Sn-2X, and Sn-3X films are all crystallized with a similar orthorhombic structure as CsFASnI_3_ (Fig. 1d)^9,31^, but the Sn-3X shows a remarkably stronger peak intensity corresponding to the (100) and (200) face than the other two samples. This proves that Sn-3X was well crystallized with high-preferred orientation in 〈X00〉 direction. The photoluminescence (PL) and time-resolved photoluminescence decay (TRPL) measurements of the corresponding three samples are shown in Supplementary Figs. 3 and 4. The highest PL signal as well as the longest carrier lifetime for Sn-3X film indicated the lowest non-radiative recombination among all three perovskite film, which would be benefit for the enhancement of *V*_oc_^13,32,33^.

### Characterizations of amorphous layer

In order to further investigate the structure of Sn-3X perovskite film, grazing incident X-ray diffraction (GIXRD) was performed (Fig. 1e). Compared to the XRD pattern of Sn-3X (Fig. 1d, blue line), no new patterns appeared with the incident angles changing from 0.5° to 0.1°, which indicates no new crystal structure was formed for Sn-3X. We then conducted the transmission electron microscopy (TEM). The sample was prepared by focused ion beam technique and protective layer of Au was deposited before ion-beam etching to exclude the effect of the environment. As can be seen in Fig. 1f, a well-crystallized Sn-3X film was covered by an amorphous layer with the thickness of 3–4 nm on it. To make it clearer, the magnified high-resolution TEM (HRTEM) image (Fig. 1f; right top) was given. Combined with the results from GIXRD and TEM, we reasoned that an amorphous layer was assembled on the surface of Sn-3X perovskite to form an amorphous-polycrystalline structure. The band gaps of Sn-3X with different content of Cl were measured by UV–visible absorption spectroscopy (Supplementary Fig. 5a–c) and the results were transformed using the Kubelka–Munk equation (Supplementary Fig. 5d–f). There is no obvious change in bandgaps of Sn-3X films (Supplementary Fig. 6), which confirms the structure of perovskite crystals was maintained when the amorphous layer was formed at the surface.

X-ray photoelectron spectroscopy (XPS) survey spectra were obtained to find out the composition of the amorphous layer (Supplementary Fig. 7a, b). According to the XPS results, the ratios of I:Sn are 1:0.58 and 1:1.47 for Sn-2X and Sn-3X, respectively, as listed in Supplementary Fig. 7c, which means a lower content of iodide was left on the Sn-3X perovskite surface. Moreover, for Sn-3X film, the atomic ratios of I:Cl and I:F were 1:0.44 and 1:0.29 (Supplementary Fig. 7d), respectively, which were much lower than 1:0.132 of I:Cl and 1:0.066 of I:F in the precursor solution of Sn-3X film. The Cl and F content at the Sn-3X perovskite surface is higher than that in precursor solution, which proves that the amorphous layer is composed of the tin triple halide of F, Cl, and I, not only a layer of SnCl_2_. As we all know, SnCl_2_ is even more hygroscopic than perovskites themselves, which will deteriorate the stability of the devices. However, the contact angle with water is enlarged from 58.9° to 89° under the existence of the amorphous layer (Supplementary Fig. 10a–c (top)), which further confirmed that it is not simply a layer of SnCl_2_. In addition, we performed the measurement of time-of-flight secondary ion mass spectroscopy (ToF-SIMS) for the Sn-3X sample to show the depth profile for the elements (Supplementary Fig. 8a). It was found that the elements of F, Cl concentrated at the surface of Sn-3X whereas I was homogeneously distributed across the film. The F and Cl quickly dropped after 8 s of sputtering time, which is consistent with the XPS results and further confirmed that the amorphous layer only existed at the surface. The element of Cl at the *x*–*y* plane (Supplementary Fig. 8b) showed uniform distribution, indicating a high coverage ratio of this amorphous layer on the surface of Sn-3X films. Otherwise, bright signals of Cl would be found.

In order to check the surface of the Sn-1X and Sn-2X films, TEM tests were also performed for the Sn-1X and Sn-2X samples (Supplementary Fig. 9); no such amorphous layer was found. The reason for the formation of the amorphous-polycrystalline structure may be the low surface energy of this amorphous layer^34,35^. The contact angles of water and toluene on the surface of Sn-1X, Sn-2X, and Sn-3X with different contents of SnCl_2_ samples were measured (Supplementary Fig. 10), and the corresponding surface energies were estimated using the Owens–Wendt model^36^. After calculation, we found that the surface energy was sharply reduced from 46.3 to 30.6 mJ m^−2^ from Sn-1X to Sn-3X samples. In addition, according to Supplementary Fig. 11a, b and the calculated surface energy values shown in Supplementary Table 1, we found that the surface energy could be further changed as the Cl content varied in Sn-3X samples, but the amount of SnF_2_ did not have this effect (Supplementary Fig. 11a, c and Table 1). Therefore, the formation of the amorphous-polycrystalline structure can be simply controlled by the amount of SnCl_2_.

### Stability of the amorphous-polycrystalline structure

**Fig. 2:**
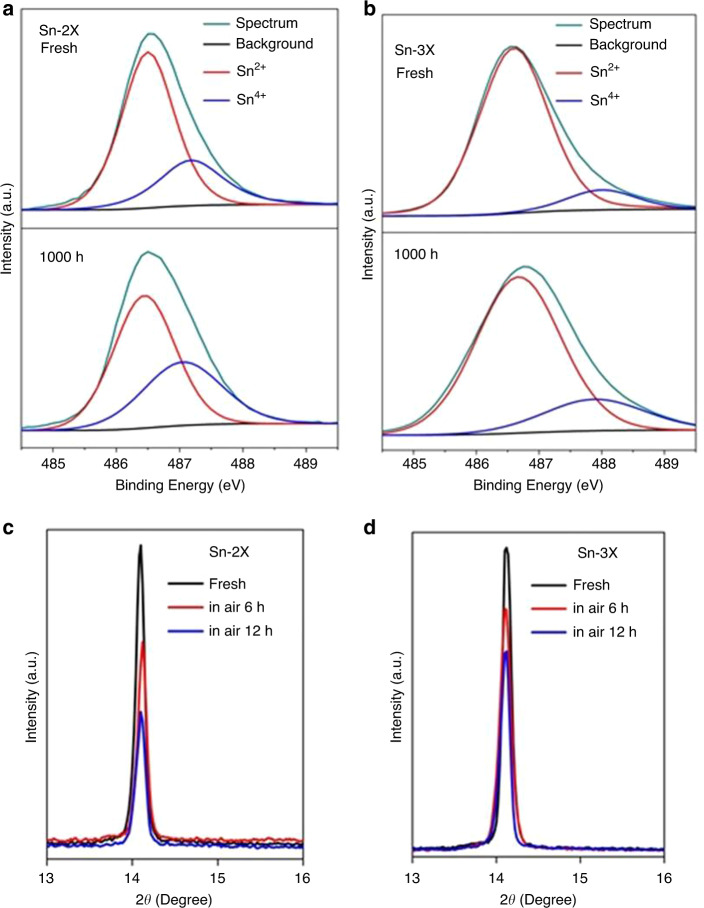
The characterization of the stability of tin perovskite layers. **a** High-resolution XPS spectrum of the Sn 3*d*_5/3_ region of Sn-2X and **b** Sn-3X film before (top) and after (bottom) being exposed to continuous simulated sunlight AM 1.5 G (100 mW cm^−2^) for 1000 h, respectively. The normalized intensity of the (100) peak in XRD patterns of **c** Sn-2X and **d** Sn-3X films as a function of time in ambient air.

The XRD patterns as a function of time were monitored to test the degradation of Sn-2X and Sn-3X films without encapsulation in ambient conditions with the humidity around 60% and temperature of 25 °C. As shown in Supplementary Fig. 12a, the aged Sn-2X film was found to show a much lower peak at 14.1° and 28.4° compared to the feature signal of fluorine-doped tin oxide substrate (FTO) at 26.5° than the fresh sample. In contrast, the feature signals of Sn-3X sample maintained the ratio to the signals of FTO (Supplementary Fig. 12b). We carefully compared the deterioration in the intensity of (100) peaks; the decay rate of Sn-2X turned out to be much faster than that of the Sn-3X sample (Fig. 2c, d), which proved the better stability of the amorphous-polycrystalline structure in Sn-3X film to oxygen and moisture. The improved stability could be attributed to the larger water contact angle of the tin triple-halide amorphous layer compared to that of Sn-2X films (Supplementary Fig. 10b, c) and its blocking of oxygen. The larger crystalline grains in Sn-3X films with fewer defects also reduced the channels for oxygen and water intrusion.

The light soaking stability of Sn-2X and Sn-3X films in N_2_ environment was also compared. XPS for Sn-2X and Sn-3X films were performed before and after aging test under AM 1.5 G simulated solar light (100 mW cm^−2^) (Fig. 2a, b). For the fresh samples, it was observed that the width of the Sn 3*d* core level in Sn-3X film was larger than that in Sn-2X, which could be ascribed to the formation of the amorphous layer in Sn-3X film. To further confirm our speculation, we etched the surface of Sn-3X with Ar ion beam to remove the amorphous layer and measured the XPS of inner perovskite (Supplementary Fig. 13); narrower Sn 3*d* signal was obtained. More importantly, the ratios of Sn^2+^ and Sn^4+^ were calculated before and after light soaking (Supplementary Fig. 14). It was found that the aged Sn-3X film had much less Sn^4+^ than Sn-2X film than the aged Sn-2X film. The stability under heat in the dark was measured by UV–visible absorption spectroscopy in N_2_. After 6 h of heating at 85 °C, Sn-2X underwent fast decay in the intensity of absorption, whereas Sn-3X maintained the absorption for 100 h (Supplementary Fig. 15a, b). The absorption decay at the wavelength of 500 nm was recorded in Supplementary Fig. 16. These results indicate the Sn-3X with this amorphous-polycrystalline structure is more stable under light and heat. The reason for the enhanced light-soaking and heat stability could be attributed to the suppressed ion migration. Devices with a structure of FTO/PEDOT:PSS/Perovskite/PCBM/BCP/Ag were fabricated based on Sn-2X and Sn-3X films and aged at the maximum power point under continuous light-soaking of AM 1.5 G (100 mW cm^−2^) and 85 °C for 100 h. XPS was used to detect the I^−^ signal in Ag electrodes (Supplementary Fig. 17); the signal of I^−^ in Ag electrode for the device based on Sn-2X is much higher than that in the device based on Sn-3X.

### Device performance

**Fig. 3:**
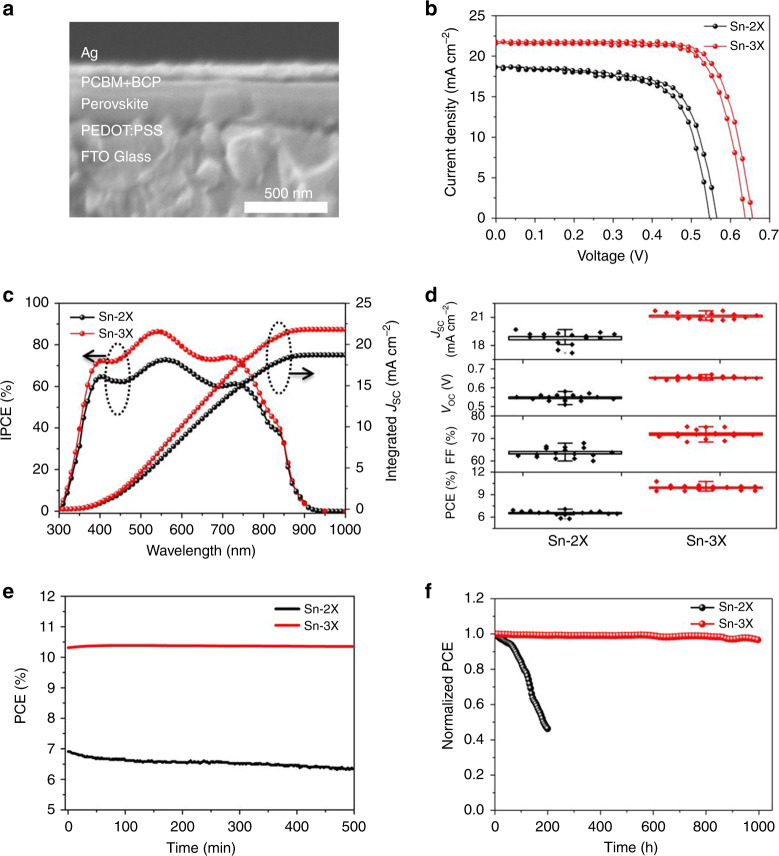
Configuration and device performance of TPSCs. **a** Cross-section SEM image of the cell based on amorphous-polycrystalline structure. **b** The *I–V* curves of Sn-2X and Sn-3X-based TPSCs. **c** The IPCE of the corresponding devices. **d** The statistical box chart of the parameters of corresponding devices and 16 cells were fabricated for each type. **e** The stabilized output of the corresponding devices for 500 min. **f** The stability test under simulated AM 1.5 G (100 mW cm^−2^) at the maximum power point. All the cells were encapsulated in N_2_ gas environment.

We then fabricated TPSC with an inverted planar structure and the cross-section SEM image of this configuration is shown in Fig. 3a. During the fabrication process, we confirmed that the amorphous-polycrystalline structure would not affect the wetting property of PCBM solution on Sn-3X film even though the contact angle was increased from 7.8° to 18° (Supplementary Fig. 18). The *I*–*V* curves of Sn-2X and Sn-3X-based cells under both scan directions are shown in Fig. 3b. The Sn-2X-based TPSC obtained an initial PCE of 6.9%, with open-circuit voltage (*V*_oc_) of 0.55 V, current density (*J*_sc_) of 18.6 mA cm^−2^, and fill factor (FF) of 67.4% under forward scan. In contrast, the PCE of Sn-3X-based TPSC was 10.4% with a *V*_oc_ of 0.64 V, a *J*_sc_ of 21.6 mA cm^−2^, and FF of 75.2%. The detailed devices parameters are summarized in Supplementary Table 2. The integrated current density of 18.7 and 21.8 mA cm^−2^ for Sn-2X-based and Sn-3X-based devices calculated form the monochromatic incident photo-to-current conversion efficiency (IPCE) (Fig. 3c) matched well with the *I*–*V* test. The *I*–*V* curves of the devices based on Sn-3X films with different amount of SnCl_2_ or the devices using only SnCl_2_ as the additives are also shown, respectively, in Supplementary Figs. 19 and 20. The improvement in device performance for Sn-3X-based devices was generally consistent across 16 devices according to the statistical box chart (Fig. 3d). To further investigate the reasons for this improvement, the band gap of this amorphous layer was measured to be 1.67 eV via electron energy loss spectroscopy (Supplementary Fig. 21). In addition, its valence band maximum (VBM) of −5.5 eV was also given by photoelectron spectroscopy (Supplementary Fig. 22b). Combined with the band gap of Sn-3X perovskite measured in Supplementary Fig. 5f and the VBM measured in Supplementary Fig. 22c, the energy level alignment of the whole device is summarized in Supplementary Fig. 23. The appropriate conduction band minimum of this amorphous layer right between those of Sn-3X perovskite and PCBM could induce less charge recombination and energy loss in devices. Dark current and transient photocurrent and photovoltage were measured to demonstrate the charge recombination and transport in a full device. As is shown in Supplementary Fig. 24, the dark current of Sn-3X-based device was three times lower than that of Sn-2X-based device. Meanwhile, the transient photovoltage results of Sn-3X-based device showed a longer decay time of 63.5 μs than the value 16.8 μs for the Sn-2X-based device (Supplementary Fig. 25a). Both dark current and transient photovoltage measurement demonstrated a lower charge recombination in a Sn-3X-based device^37,38^ In addition, Sn-3X-based cells showed faster decay in transient photocurrent with a lifetime of 1.37 μs compared to the 3.45 μs of the Sn-2X-based cell (Supplementary Fig. 25b), indicating better charge transport in Sn-3X-based devices^39,40^. The corresponding stabilized power output of Sn-2X and Sn-3X are shown in Fig. 3e, where Sn-3X presented a constant stabilized output efficiency of about 10.3% with an applied voltage of 0.52 V for 500 min. In contrast, Sn-2X-based cell tended to exhibit a slow decrease in stabilized output during the whole test. Furthermore, the Sn-3X cells encapsulated in a N_2_ glove box maintained over 95% of its initial PCE after operation at the maximum power point under simulated illumination of AM 1.5 G (100 mW cm^−2^) for 1000 h. In contrast, Sn-2X-based cell lost more than 50% of PCE within 200 h (Fig. 3f). One of our typical Sn-3X-based cells was sent to an accredited test center (Newport, USA); a certified efficiency of 10.08% was obtained with a *V*_oc_ of 0.64 V, *J*_sc_ of 22.2 mA cm^−2^ and FF of 70.8% (Supplementary Fig. 26). The certified results of *I*–*V* curve (Supplementary Fig. 27) and normalized external quantum efficiency (EQE) (Supplementary Fig. 28) were also provided. More importantly, it should be noted that the certified efficiency obtained here is a quasi-steady-state efficiency, which means that every point in the *I*–*V* curve will not be obtained until the current can be unchanging at a level within 0.03% under a constant applied voltage, indicating the reliable working stability of TPSC based on this amorphous-polycrystalline structure.

## Discussion

In this work, we fabricated a TPSC with an amorphous-polycrystalline structure, which enabled high efficiency and stable TPSCs due to its suitable energy level and blocking effect on ion diffusion, oxygen, and moisture. The certified quasi-steady-state efficiency over 10% for TPSCs was achieved from an accredited center. Our work indicates a way to build efficient and stable TPSCs with reliable measurement results.

## Methods

### Materials

The following chemicals were used as received from commercial sources, including SnI_2_ (99.99%, Sigma-Aldrich), CsI (99.9%, Sigma-Aldrich), CH(NH_2_)_2_I (FAI) (>98%, Tokyo Chemical Industry Co., Japan), SnF_2_ (>99%, Sigma-Aldrich), SnCl_2_(>99%, Sigma-Aldrich), PCBM (99.5% Lumtec Co., Taiwan), bathocuproine (>99%, Wako), ethylenediammonium diiodide (EDAI_2_) (>98%, Tokyo Chemical Industry Co., Japan). All solutions were filtered with 0.2 μm PTFE filter before use.

### Solar cell fabrication

FTO glass substrates were patterned and cleaned by detergent, DI water, acetone, and isopropanol for 20 min via sonication treatment, respectively. Then, the substrates were treated by ultraviolet-ozone for 30 min before the deposition of PEDOT:PSS. For CsFASnI_3_ perovskite precursor solution, CsI, FAI, SnI_2_, SnF_2_, EDAI_2_ with a molar ratio of 0.2:0.8:1:0.1:0.01 were dissolved in DMSO solvent with a total concentration of 0.9 M^13,33,41^. Different concentrations of SnCl_2_ were added to the CsFASnI_3_ precursor solution to study their effect. (The molar ratio of cesium was fixed to 20 mol% according to previous work.)^42^. The perovskite films were spin-coated on the PEDOT:PSS layer at 1000 rpm for 12 s, and 5000 rpm for 40 s in the glove box. 150 μl of chlorobenzene was in-situ dripped onto the perovskite film after 30 s during the second step. Afterward, the perovskite films were annealed at 60 °C for 5 s and 100 °C for 15 min. PCBM (20 mg/ml in chlorobenzene) solution was spin-coated onto the perovskite film at 1000 rpm for 60 s and 5000 rpm for 5 s. Finally, 8 nm BCP and 100 nm Ag electrode were evaporated under high vacuum (<2 × 10^−7^ Torr). The device area was defined using masks as 0.09 cm^2^, respectively. The devices were fabricated in a glove box filled with N_2_ and the oxygen concentration was always kept under 0.1 ppm. Finally, the solar cells were encapsulated in N_2_-filled glove box by cavity glass with UV glue on the edges. The encapsulation was finished after 10 s of soaking under UV light and the photographs of our encapsulated device are provided in Supplementary Fig. 29. The solar cells sent to the certification institute were further packaged in a black cooling system to keep the temperature around 25 °C.

### Characterization

The SEM images were measured by JSM-6500F field-emission scanning electron microscope. The XRD was measured by Rigaku RINT-2500 powder X-ray diffractometer using Cu K_α_ radiation. The transmission electron microscope (TEM), high-resolution transmission electron microscope (HRTEM), and electron energy loss spectroscopy were obtained by a JEM-2100F field emission electron microscope. To prepare TEM sample by focused ion beam (JEM-9320FIB), the protective layers of Au were deposited before ion-beam etching. To prepare other TEM samples, the perovskite films were removed from FTO glass to Cu grid in a nitrogen-filled glovebox and transformed to TEM chamber with the protection of N_2_ gas to prevent the possible oxidation of the film. The UV–vis spectra were obtained by a Shimadzu UV/vis 3600 spectrophotometer. The photoelectron spectroscopies were measured by a photoelectron spectrometer (Riken-keiki AC-3). The PL and TRPL decay were measured with a Hamamatsu fluorescence spectrometer. The XPS spectra were measured by PHI Quantera SXM (ULVAC-PHI) with X-ray source of Al Kα (mono), the incident angle and take off angle are 90° and 45°, respectively. Surface energy was estimated using the Owens–Wendt model^36^, the equations are as follows:

1 $${\it{r}}_{\mathrm{{{s}}}} = r_{\mathrm{{{s}}}}^{\mathrm{{{D}}}} + r_{\mathrm{{{s}}}}^{\mathrm{{{P}}}}$$

2 $${\it{r}}_{\mathrm{{{L}}}} = {\it{r}}_{\mathrm{{{L}}}}^{\mathrm{{{D}}}} + {\it{r}}_{\mathrm{{{L}}}}^{\mathrm{{{P}}}}$$

3 $${\it{r}}_{\mathrm{{{L}}}}\left( {1 + {\mathrm{{cos}}}\,\theta } \right) = 2\left( {{\it{r}}_{\mathrm{{{s}}}}^{\mathrm{{{D}}}}{\it{r}}_{\mathrm{{{L}}}}^{\mathrm{{{D}}}}} \right)^{1/2} \,+ \, 2\left( {{\it{r}}_{\mathrm{{{s}}}}^{\mathrm{{{P}}}}{\it{r}}_{\mathrm{{{s}}}}^{\mathrm{{{P}}}}} \right)^{1/2}$$

In Eq. (1), *r*_s_ is the overall surface energy of the solid, ${\it{r}}_{\mathrm{{{s}}}}^{\mathrm{{{D}}}}$ is the dispersive component of the surface tension of the solid, ${\it{r}}_{\mathrm{{{s}}}}^{\mathrm{{{P}}}}$ is the polar component of the surface tension of the solid. In Eq. (2), *r*_L_ is the overall surface tension of the wetting liquid, ${\it{r}}_{\mathrm{{{L}}}}^{\mathrm{{{D}}}}$ is the dispersive component of the surface tension of the wetting liquid, ${\it{r}}_{\mathrm{{{L}}}}^{\mathrm{{{P}}}}$ is the polar component of the wetting liquid. In Eq. (3), *θ* is the contact angle of liquid on a solid surface. Water $\left( {{\mathrm{{{r}}}}_{\mathrm{{{L}}}}^{\mathrm{{{D}}}} = 21.8\,{\mathrm{{mJ}}}\,{\mathrm{{m}}}^{ - 2}, {\it{r}}_{\mathrm{{{s}}}}^{\mathrm{{{P}}}} = 51.0\,{\mathrm{{mJ}}}\,{\mathrm{{m}}}^{ - 2}} \right)$ and Toluene $\left( {{\mathrm{{{r}}}}_{\mathrm{{{L}}}}^{\mathrm{{{D}}}} = 28.4\,{\mathrm{{mJ}}}\,{\mathrm{{m}}}^{ - 2}, {\it{r}}_{\mathrm{{{s}}}}^{\mathrm{{{P}}}} = 0\,{\mathrm{{mJ}}}\,{\mathrm{{m}}}^{ - 2}} \right)$ were used as tested liquid. The *J–V* curves were measured under forward scan (−0.1 to 0.7 V) or reverse scan (0.7 to −0.1 V) with a fixed step voltage of 10 mV and delay time of 50 ms by a solar simulator with standard air mass 1.5 sunlight (100 mW cm^−2^, WXS-155S-10, Wacom Denso) in air at room temperature near 25 °C according to our previous report^43,44^. We used a reference cell, which was calibrated by the Calibration, Standards and Measurement Team at the Research Center for Photovoltaics in AIST, Japan. The spectral mismatch is <3%. The aperture area was defined by a mask of 0.09 cm^2^. Monochromatic IPCE spectra were measured by a monochromatic incident light of 1 × 10^16^ photons cm^−2^ in director current mode (CEP-2000BX, Bunko-Keiki). The light intensity of the solar simulator was calibrated by a standard silicon solar cell. The operational stability was tested on a solar cell light resistance test system (Model BIR-50, Bunko-keiki) with a Class AAA solar simulator. Then the operational stability was tested on a solar cell light resistance test system (Model BIR-50, Bunko-keiki) with a Class AAA solar simulator. All the tests except for the stability test in ambient air are using the encapsulated cells or the samples kept in boxes filled with N_2_, and transferred using those boxes too to exclude the formation of Sn-oxide^45^. The cells used for the ToF-SIMS tests are encapsulated first to undergo the aging test and then were unpackaged to test the ToF-SIMS.

### Reporting summary

Further information on research design is available in the Nature Research Reporting Summary linked to this article.

## Supplementary information

Supplementary Information

Solar Cells Reporting Summary

## Data Availability

The data that support the findings of this study are available from the corresponding authors on reasonable request.
