# Supplementary material for: Efficient and stable tin perovskite solar cells enabled by amorphous-polycrystalline structure
Source: Nat Commun. 2020 May 29;11:2678. doi: 10.1038/s41467-020-16561-6 (PMC7260362; doi:10.1038/s41467-020-16561-6)
Supplement: Supplementary file 1 — Supplementary Information [file 41467_2020_16561_MOESM1_ESM.pdf]

Supplementary Information

Efficient and Stable Tin Perovskite Solar Cells Enabled by Amorphous-polycrystalline Structure

Liu et al.

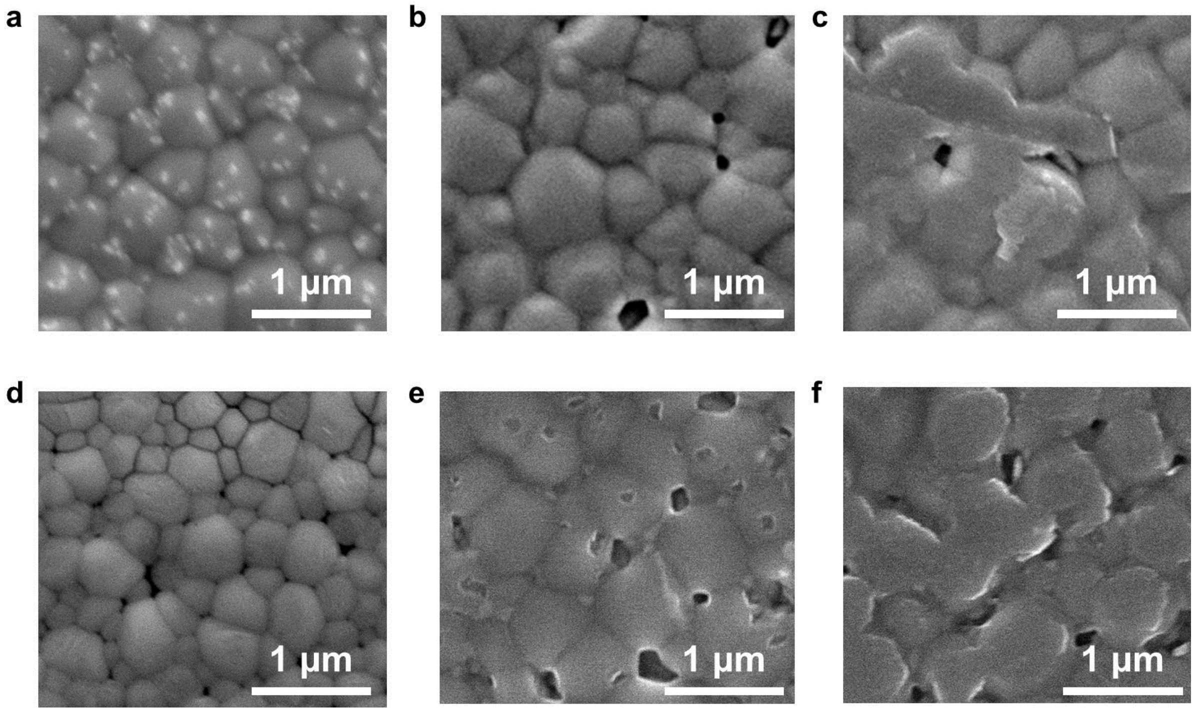

Supplementary Figure 1. SEM images of tin perovskite films with different amount of additives. **(a)** $CsFASnI_3$-20%SnF_2, **(b)** $CsFASnI_3$-10%SnF_2-10%$SnCl_2$, **(c)** $CsFASnI_3$-10%SnF_2-30%$SnCl_2$, **(d)** $CsFASnI_3$-10%$SnCl_2$, **(e)** $CsFASnI_3$-20%$SnCl_2$, **(d)** $CsFASnI_3$-30%$SnCl_2$.

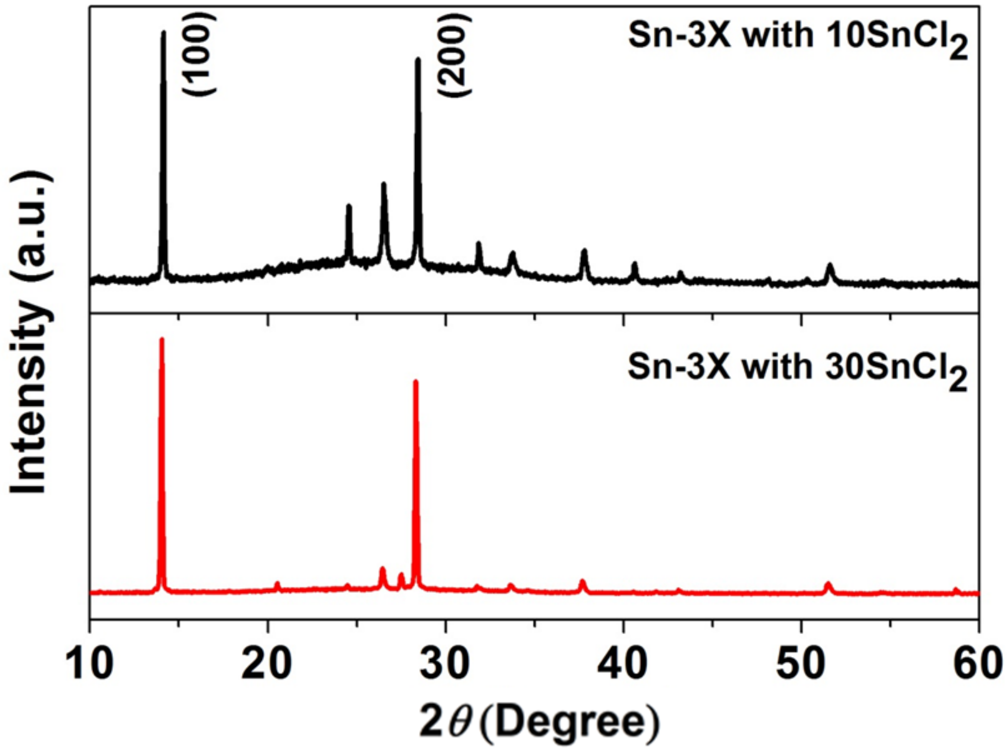

Supplementary Figure 2. XRD patterns of Sn-3X films with varied Cl content.

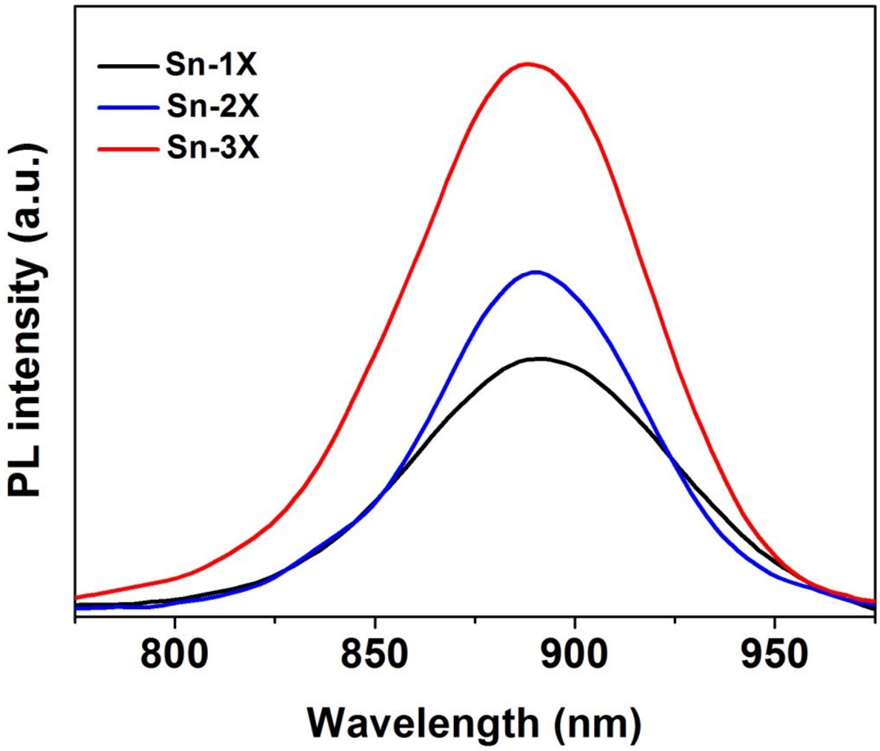

Supplementary Figure 3. PL spectra of tin perovskite films.

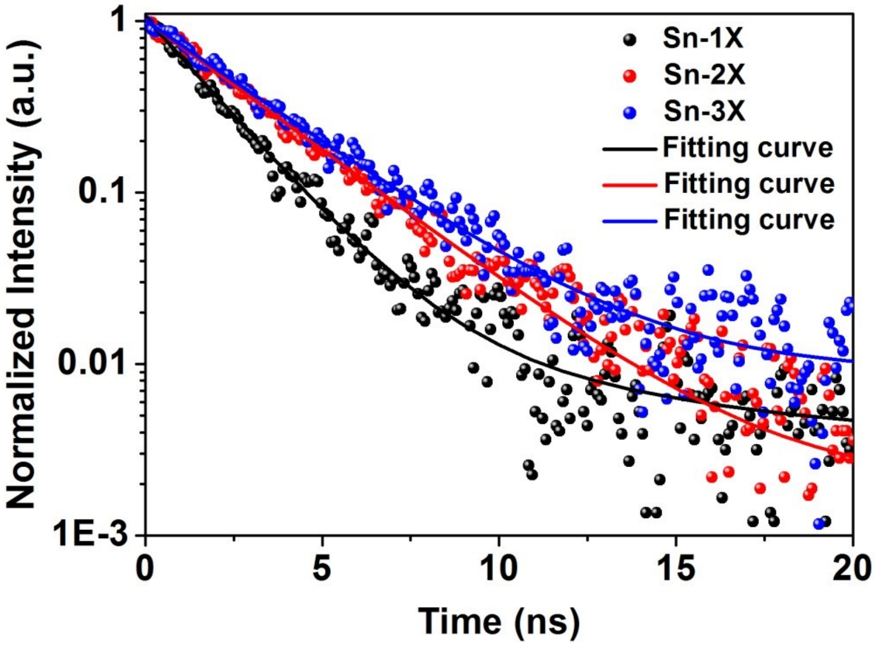

Supplementary Figure 4. TRPL spectra of tin perovskite films.

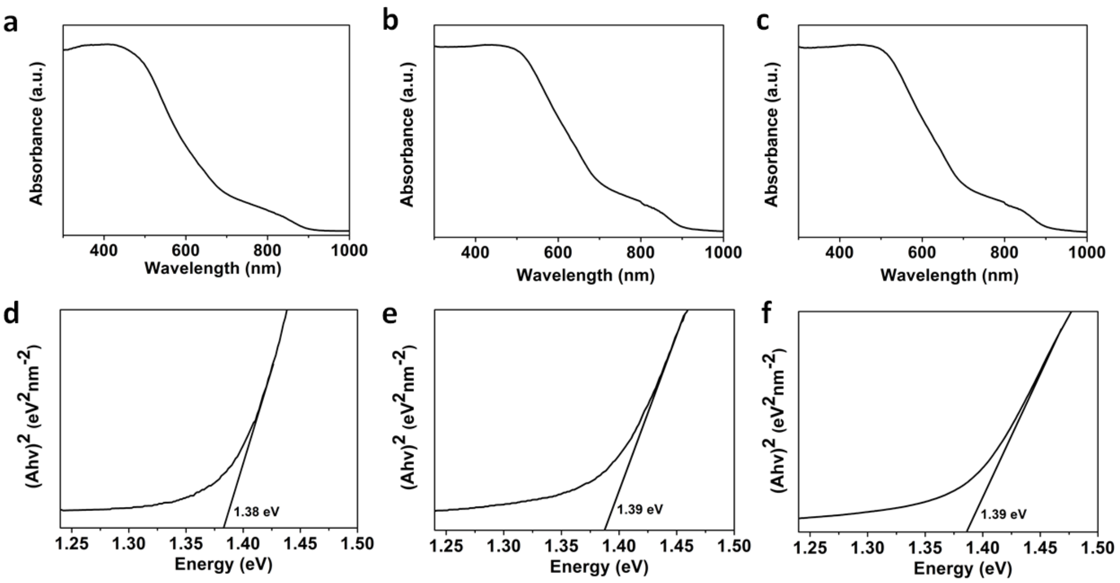

Supplementary Figure 5. Band gap measurement of perovskite films with increasing Cl content. UV-vis absorption spectra of **(a)** Sn-3X with 0 mol% $SnCl_2$, **(b)** Sn-3X with 10 mol% $SnCl_2$ and **(c)** Sn-3X with 20 mol% $SnCl_2$ perovskite films. **(d-f)** The corresponding Kubelka–Munk-transformed diffuse reflectance spectrum of the three samples.

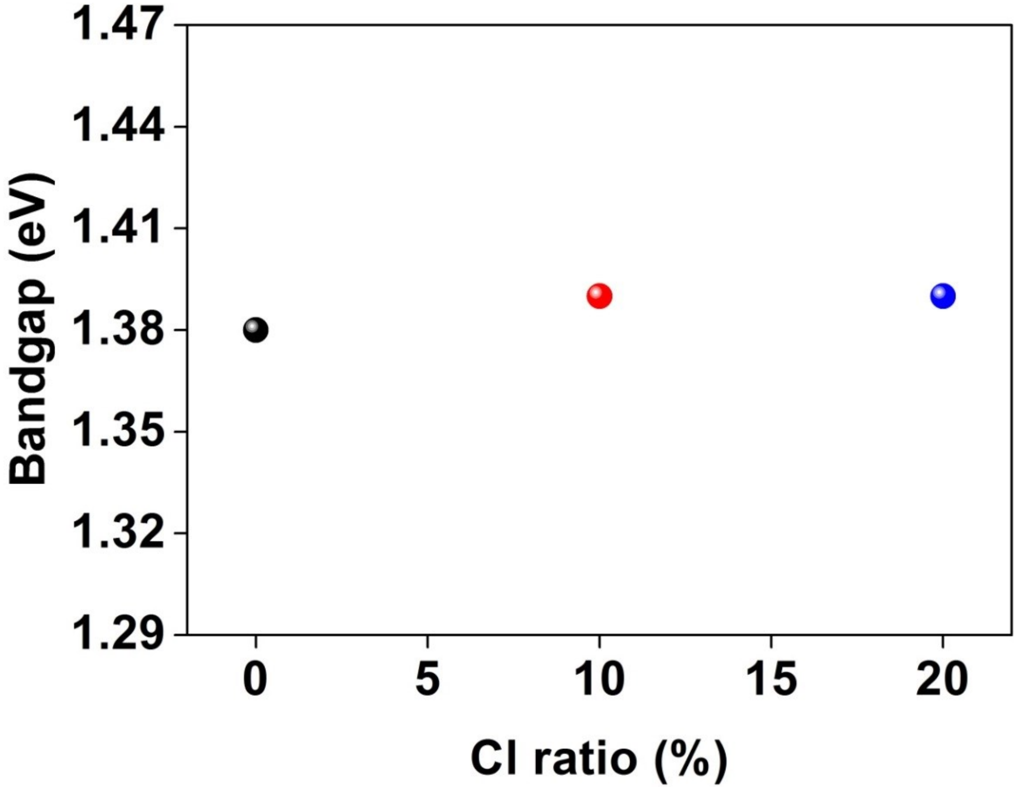

Supplementary Figure 6. Bandgaps of Sn-3X with the increase in Cl content from 0% to 20 mol%.

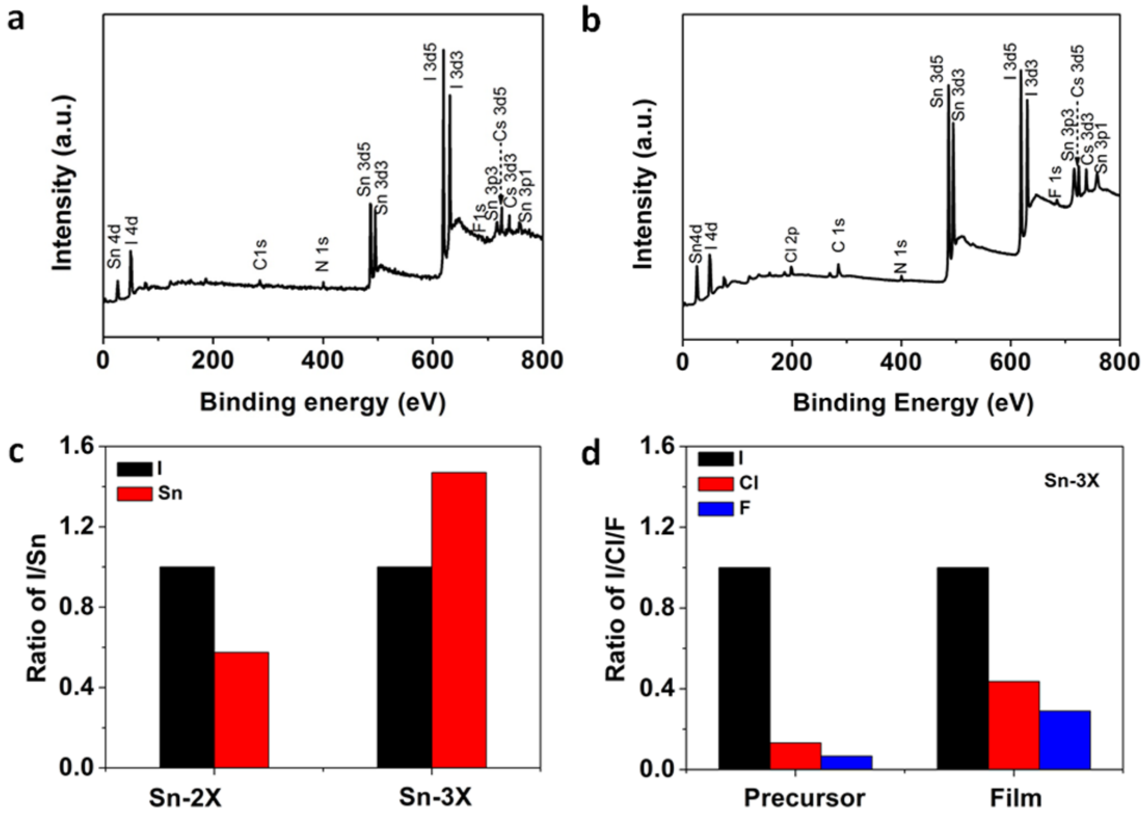

Supplementary Figure 7. The composition of the amorphous layer. XPS spectra of **(a)** Sn-2X and **(b)** Sn-3X perovskite films. **(c)** The I/Sn ratio of Sn-2X and Sn-3X perovskite obtained from XPS results. **(d)** The I/Cl/F ratio of Sn-3X precursor solution and Sn-3X perovskite film obtained from XPS results.

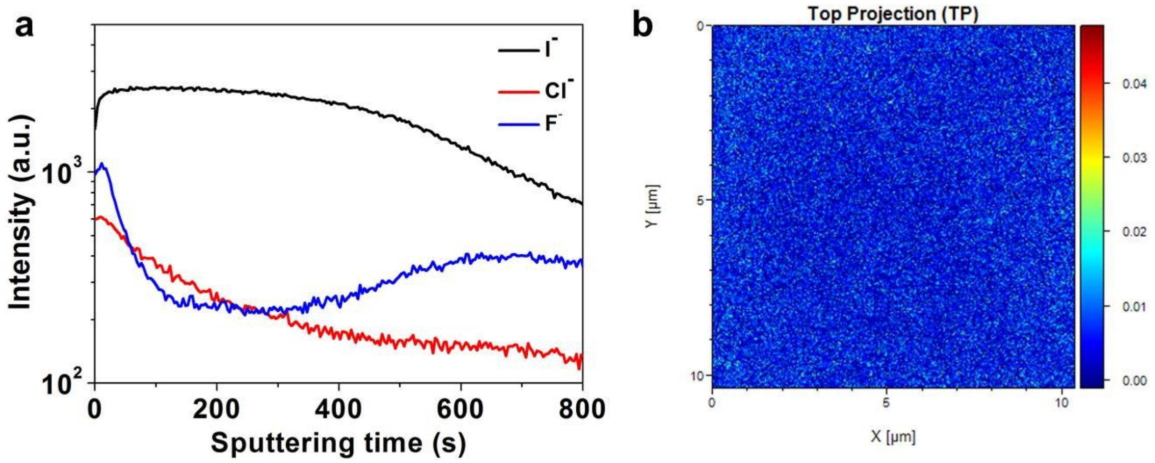

Supplementary Figure 8. The ToF-SIMS of Sn-3X film to show the element depth and x-y plane profile. (a) The elements depth profile of Sn-3X film, (b) the distribution of Cl in x-y plane in Sn-3X film.

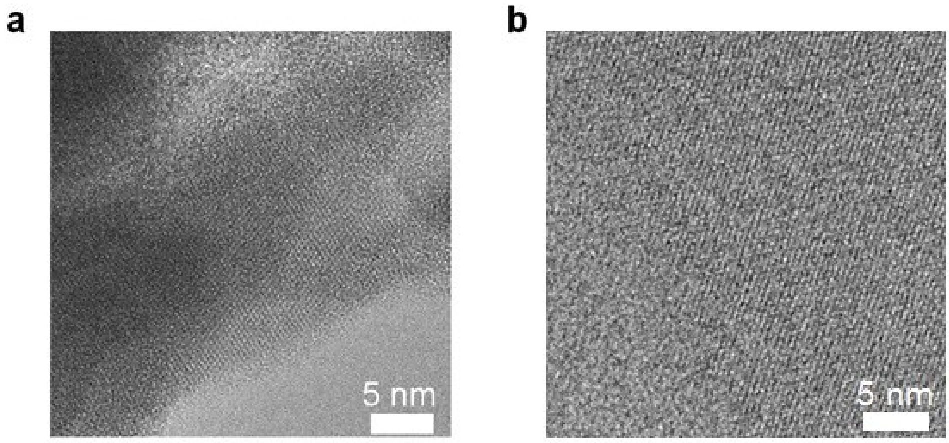

Supplementary Figure 9. The proof for the inexistence of the amorphous layer on Sn-1X and Sn-2X films. The TEM images of (a) Sn-1X, (b) Sn-2X films.

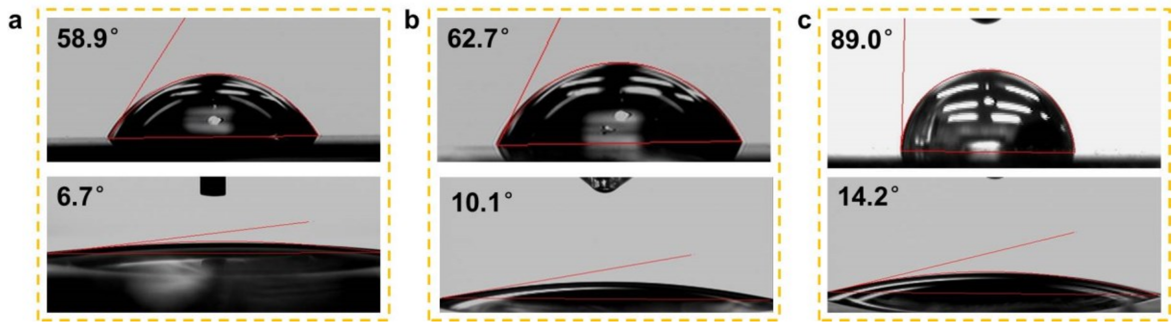

Supplementary Figure 10. The reason for the formation of the amorphous-polycrystalline structure in Sn-3X film. The contact angles of water and toluene on (a) Sn-1X, (b) Sn-2X and (c) Sn-3X films.

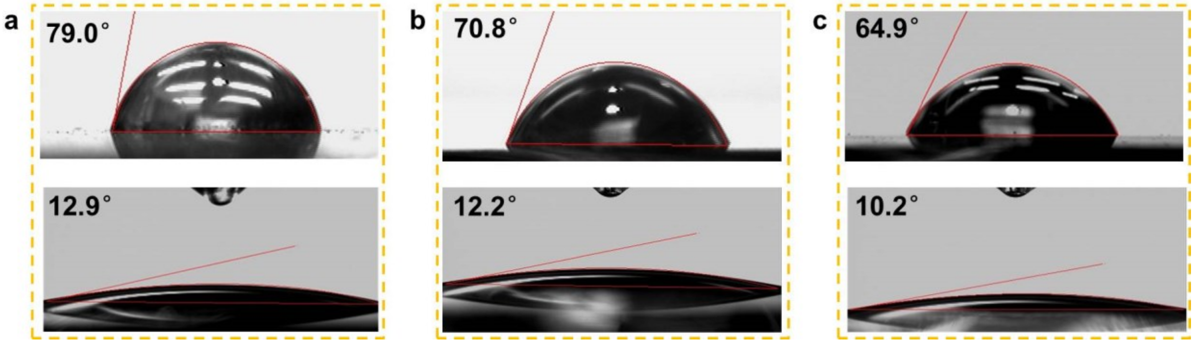

Supplementary Figure 11. The reason for the formation of the amorphous-polycrystalline structure in Sn-3X film. The contact angles of water and toluene on **(a)** $CsFASnI_3$-10%SnF_2-10%$SnCl_2$, **(b)** $CsFASnI_3$-10%SnF_2-30%$SnCl_2$, **(c)** $CsFASnI_3$-20%SnF_2.

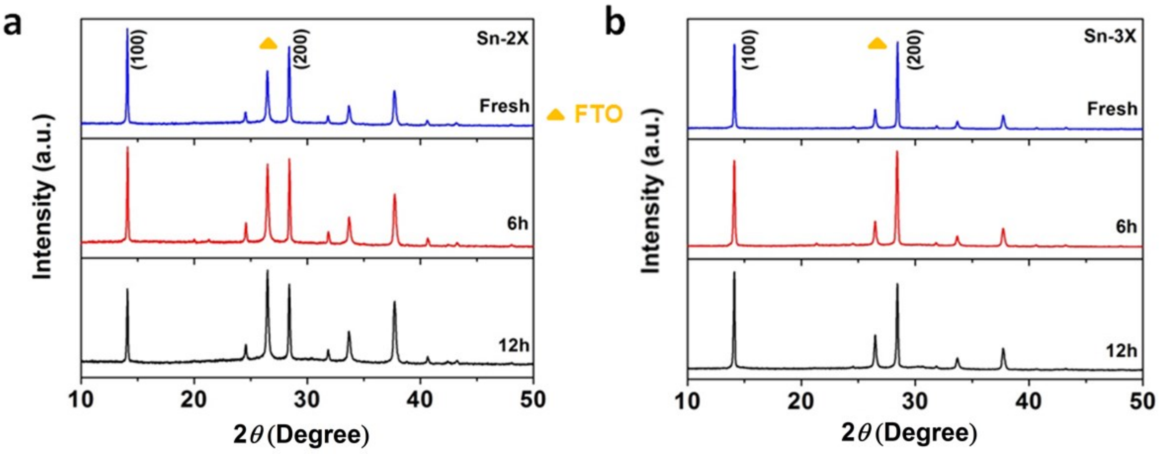

Supplementary Figure 12. Stability of the amorphous-polycrystalline structure in ambient air. XRD patterns of **(a)** Sn-2X and **(b)** Sn-3X as a function of time in ambient air with the humidity around 60% and temperature of 25 ^{o}C.

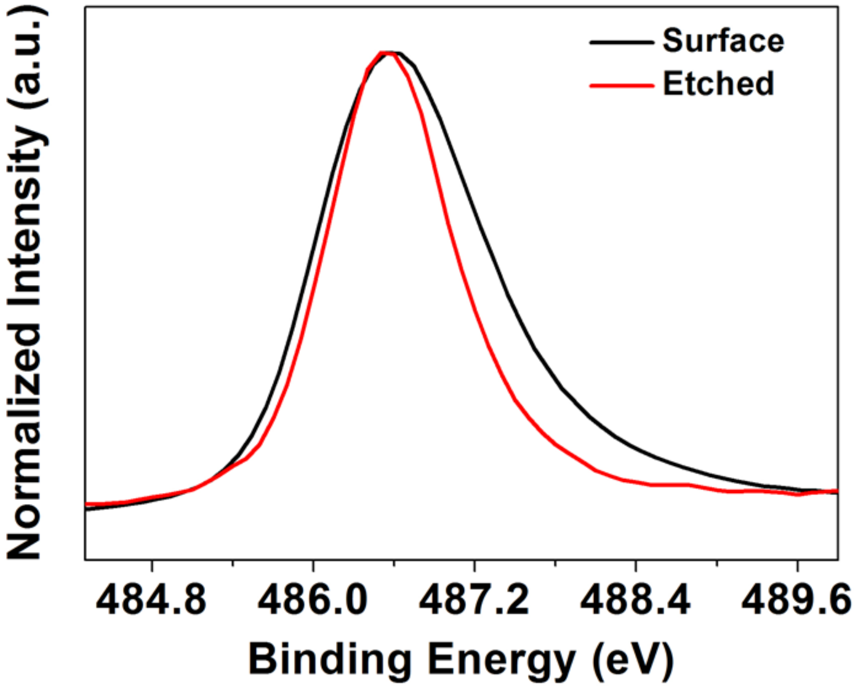

Supplementary Figure 13. XPS results of Sn-3X film from the top to the inner side.

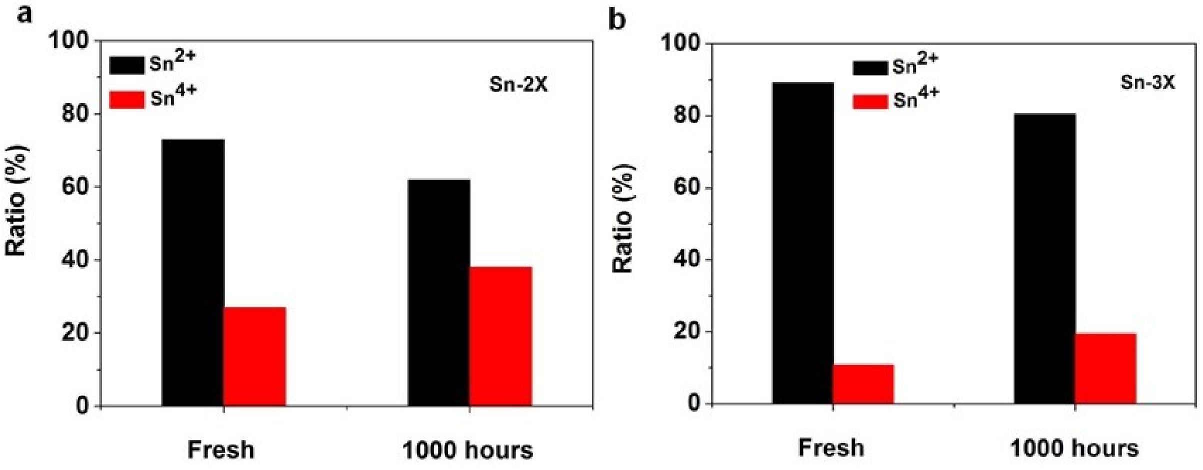

Supplementary Figure 14. Light-soaking Stability of the amorphous-polycrystalline structure in N_2. The Sn^{4+} and Sn^{2+} ratio statistic histogram of the XPS results in Figures 2a and b.

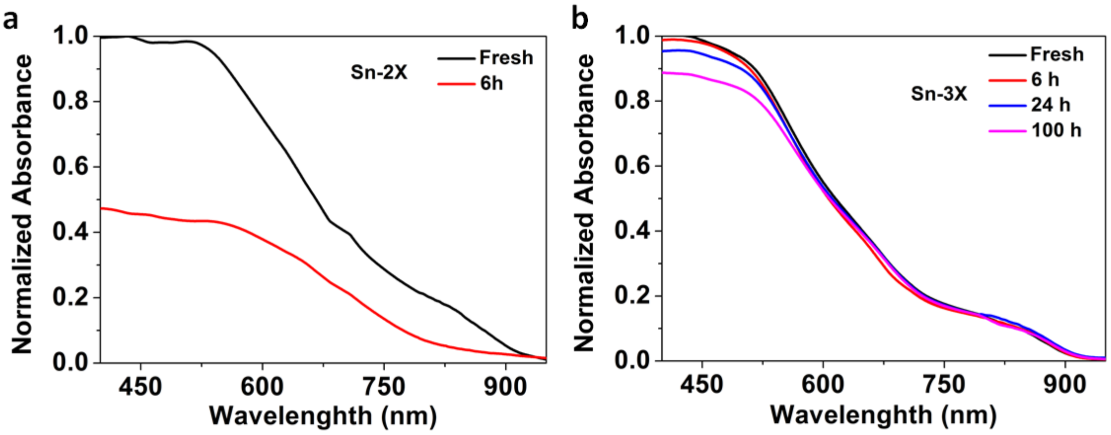

Supplementary Figure 15. Stability of the amorphous-polycrystalline structure under heat. UV-vis spectra of **(a)** Sn-2X and **(b)** Sn-3X as a function of time heated at the temperature of 85 ^{o}C in the dark in N_2.

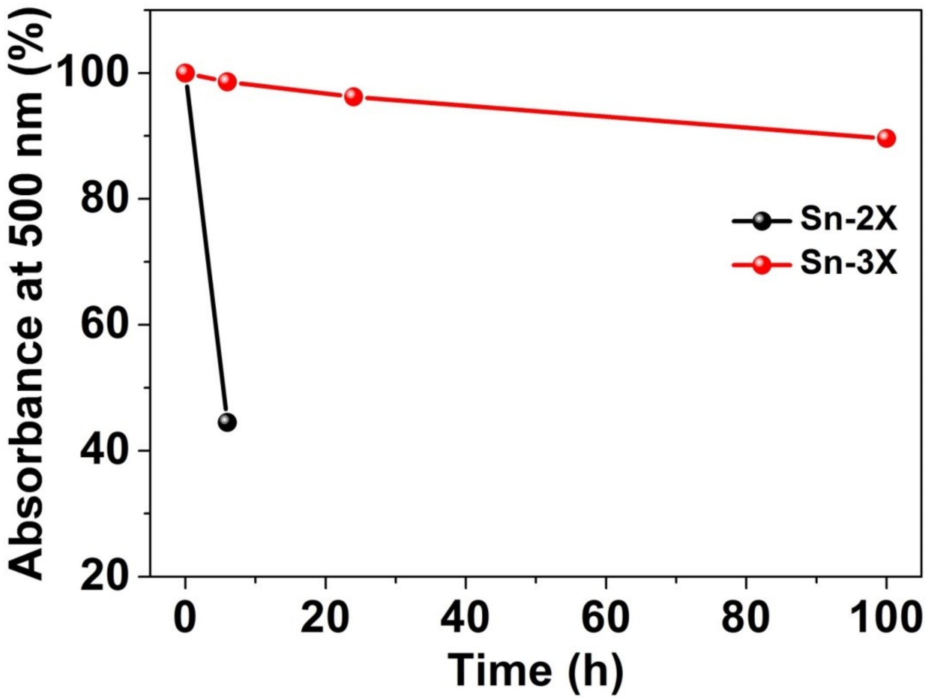

Supplementary Figure 16. Stability of the amorphous-polycrystalline structure under heat. Absorbance at 500 nm as a function of time summarized from the data in Supplementary Figure 15.

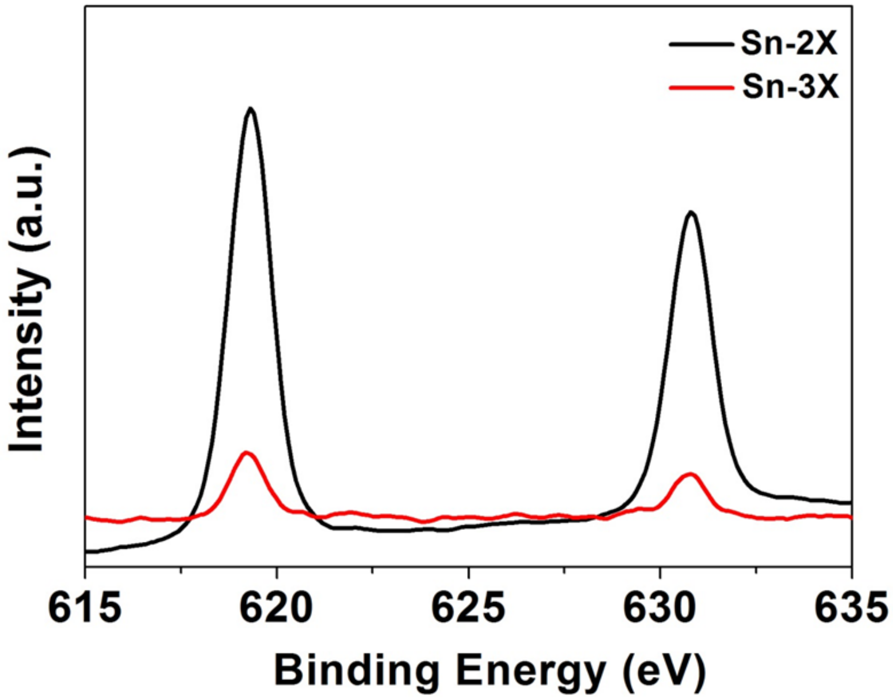

Supplementary Figure 17. The suppression of ion diffusion in amorphous-polycrystalline structured devices. The XPS results of I 3*d* core levels in the Ag electrodes of Sn-2X and Sn-3X based devices.

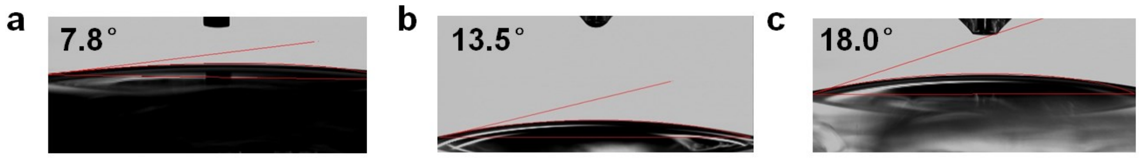

Supplementary Figure 18. The wetting property of PCBM solution on the tin perovskite films. The contact angles of PCBM solution on the surface of (a) Sn-1X, (b) Sn-2X and (c) Sn-3X films.

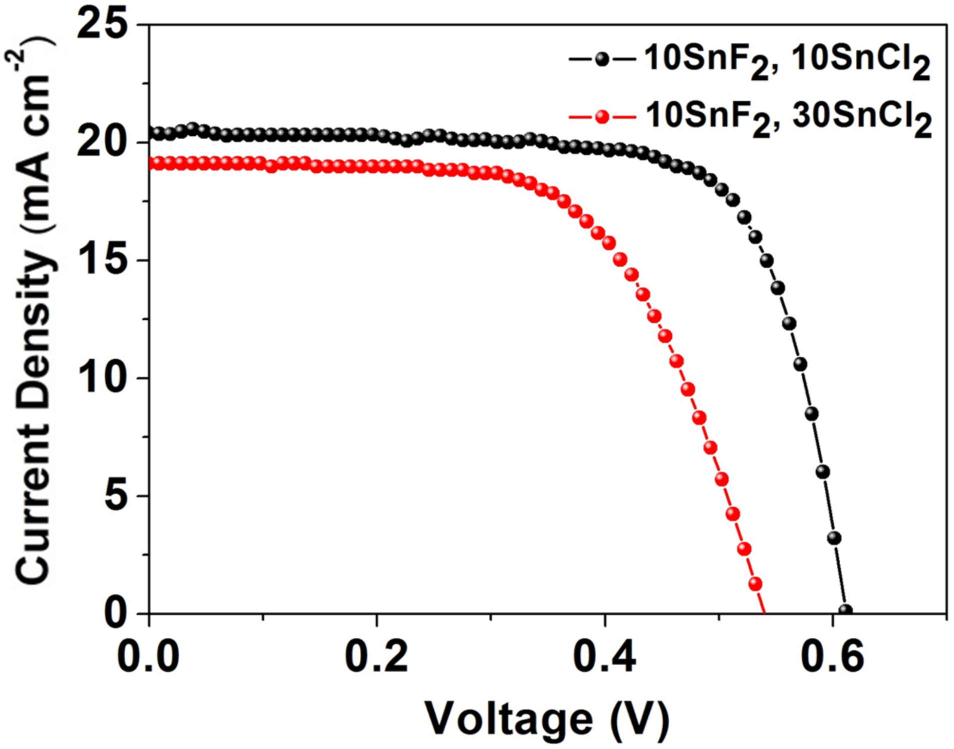

Supplementary Figure 19. The *I-V* curves of Sn-3X based devices with different amount of $SnCl_2$.

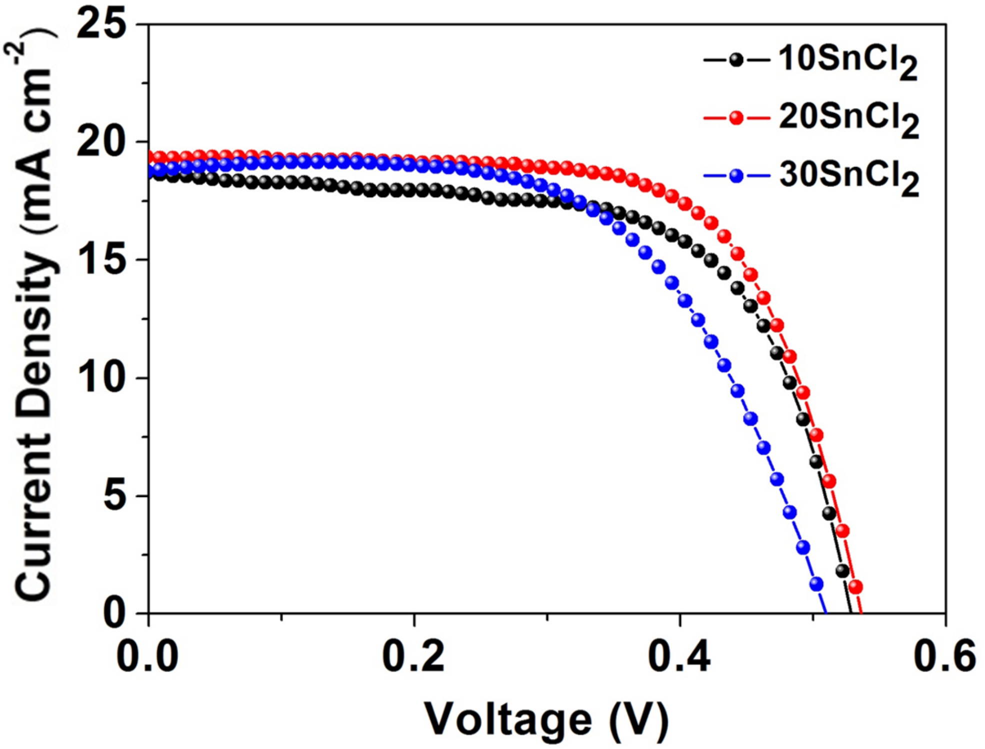

Supplementary Figure 20. The *I-V* curves of devices using only $SnCl_2$ as the additive.

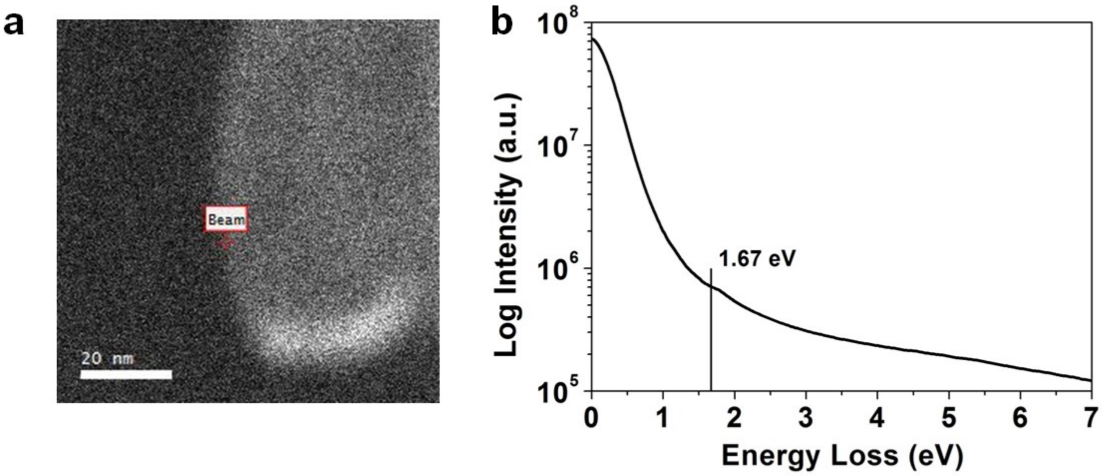

Supplementary Figure 21. Band gap measurement of the amorphous layer. (a) The TEM image of Sn-3X sample, (b) the electron energy loss spectroscopy of the edge of the sample, denotes as beam in Supplementary Figure 21a.

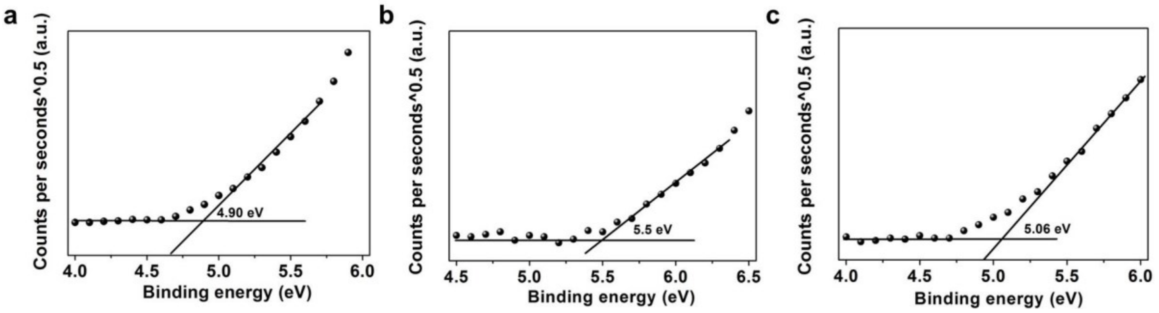

Supplementary Figure 22. VBM measurement of Sn-2X, the amorphous layer and Sn-3X perovskite. The photoelectron spectroscopies of (a) Sn-2X film, (b) the amorphous layer and (c) Sn-3X film etched with Ar ion beam.

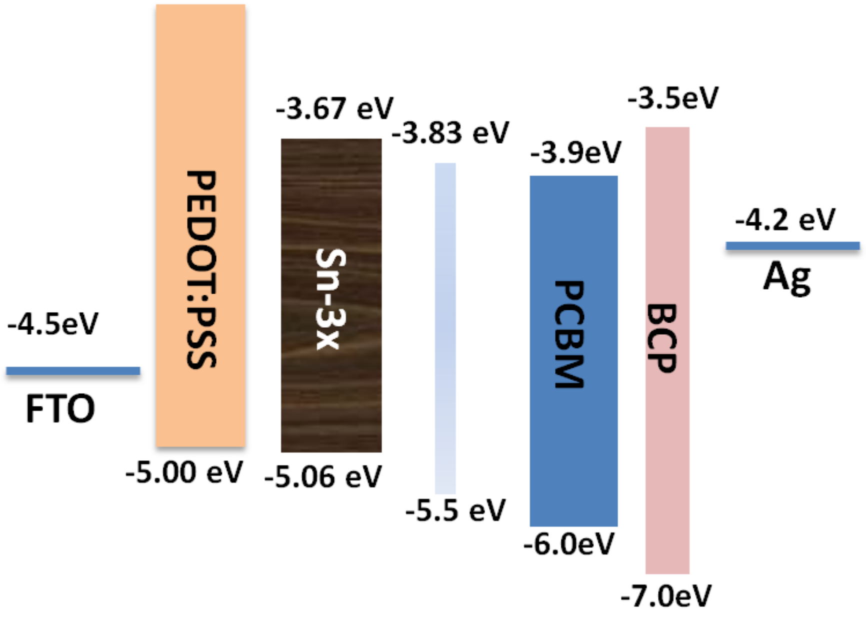

Supplementary Figure 23. The energy level alignment of the amorphous-polycrystalline structured devices.

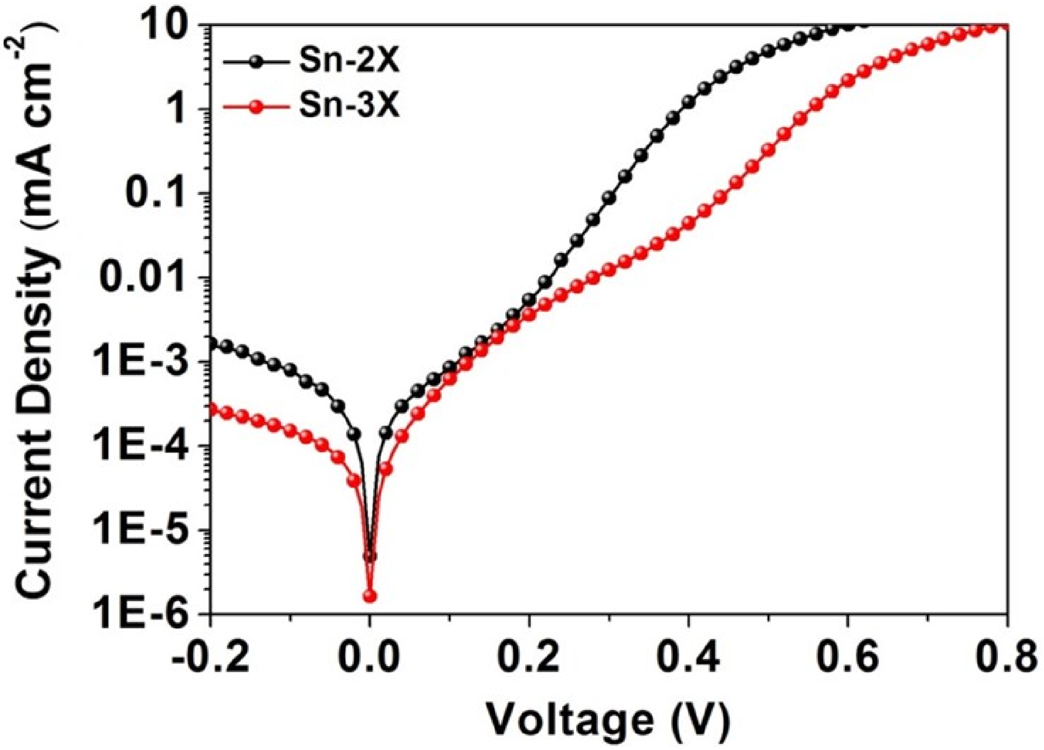

Supplementary Figure 24. Investigation of charge recombination in devices. The dark current of Sn-2X and Sn-3X based devices.

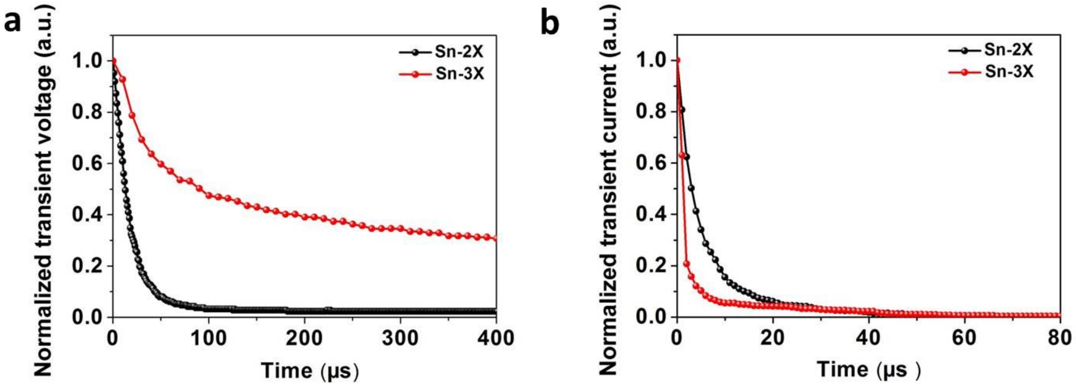

Supplementary Figure 25. Investigation of charge extraction and transport in devices. **(a)** Normalized transient photovoltage and **(b)** Normalized transient photocurrent of Sn-2X and Sn-3X based cells.

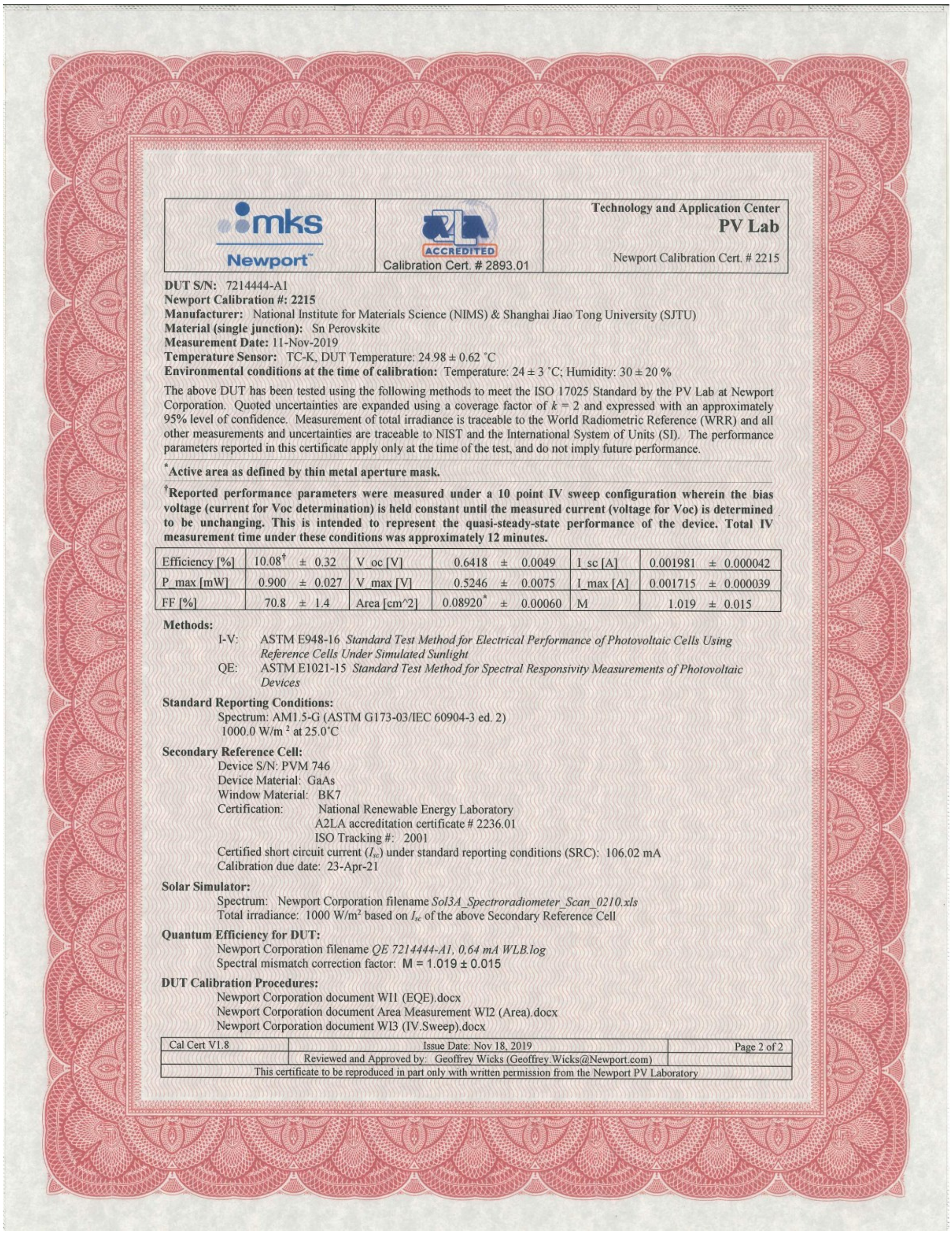

mks Newport	ACCREDITED Calibration Cert. # 2893.01	**Technology and Application Center** **PV Lab** Newport Calibration Cert. # 2215

DUT S/N: 7214444-A1
Newport Calibration #: 2215
Manufacturer: National Institute for Materials Science (NIMS) & Shanghai Jiao Tong University (SJTU)
Material (single junction): Sn Perovskite
Measurement Date: 11-Nov-2019
Temperature Sensor: TC-K, DUT Temperature: 24.98 ± 0.62 °C
Environmental conditions at the time of calibration: Temperature: 24 ± 3 °C; Humidity: 30 ± 20 %

The above DUT has been tested using the following methods to meet the ISO 17025 Standard by the PV Lab at Newport Corporation. Quoted uncertainties are expanded using a coverage factor of $k = 2$ and expressed with an approximately 95% level of confidence. Measurement of total irradiance is traceable to the World Radiometric Reference (WRR) and all other measurements and uncertainties are traceable to NIST and the International System of Units (SI). The performance parameters reported in this certificate apply only at the time of the test, and do not imply future performance.

***Active area as defined by thin metal aperture mask.**

†Reported performance parameters were measured under a 10 point IV sweep configuration wherein the bias voltage (current for Voc determination) is held constant until the measured current (voltage for Voc) is determined to be unchanging. This is intended to represent the quasi-steady-state performance of the device. Total IV measurement time under these conditions was approximately 12 minutes.

Efficiency [%]	10.08† ± 0.32	V_oc [V]	0.6418 ± 0.0049	I_sc [A]	0.001981 ± 0.000042
P_max [mW]	0.900 ± 0.027	V_max [V]	0.5246 ± 0.0075	I_max [A]	0.001715 ± 0.000039
FF [%]	70.8 ± 1.4	Area [cm^2]	0.08920* ± 0.00060	M	1.019 ± 0.015

Methods:
I-V: ASTM E948-16 *Standard Test Method for Electrical Performance of Photovoltaic Cells Using Reference Cells Under Simulated Sunlight*
QE: ASTM E1021-15 *Standard Test Method for Spectral Responsivity Measurements of Photovoltaic Devices*

Standard Reporting Conditions:
Spectrum: AM1.5-G (ASTM G173-03/IEC 60904-3 ed. 2)
1000.0 W/m^2 at 25.0°C

Secondary Reference Cell:
Device S/N: PVM 746
Device Material: GaAs
Window Material: BK7
Certification: National Renewable Energy Laboratory
A2LA accreditation certificate # 2236.01
ISO Tracking #: 2001
Certified short circuit current (I_{sc}) under standard reporting conditions (SRC): 106.02 mA
Calibration due date: 23-Apr-21

Solar Simulator:
Spectrum: Newport Corporation filename *Sol3A_Spectroradiometer_Scan_0210.xls*
Total irradiance: 1000 W/m^2 based on I_{sc} of the above Secondary Reference Cell

Quantum Efficiency for DUT:
Newport Corporation filename *QE 7214444-A1, 0,64 mA WLB.log*
Spectral mismatch correction factor: M = 1.019 ± 0.015

DUT Calibration Procedures:
Newport Corporation document WI1 (EQE).docx
Newport Corporation document Area Measurement WI2 (Area).docx
Newport Corporation document WI3 (IV.Sweep).docx

Cal Cert V1.8	Issue Date: Nov 18, 2019	Page 2 of 2
	Reviewed and Approved by: Geoffrey Wicks (Geoffrey.Wicks@Newport.com)	
This certificate to be reproduced in part only with written permission from the Newport PV Laboratory		

Supplementary Figure 26. The certified results of a typical amorphous-polycrystalline structured TPSC obtained from an accredited photovoltaic certification institute (Newport，USA). A quasi-steady-state PCE of 10.08% was obtained on an aperture area of 0.08920 cm^2.

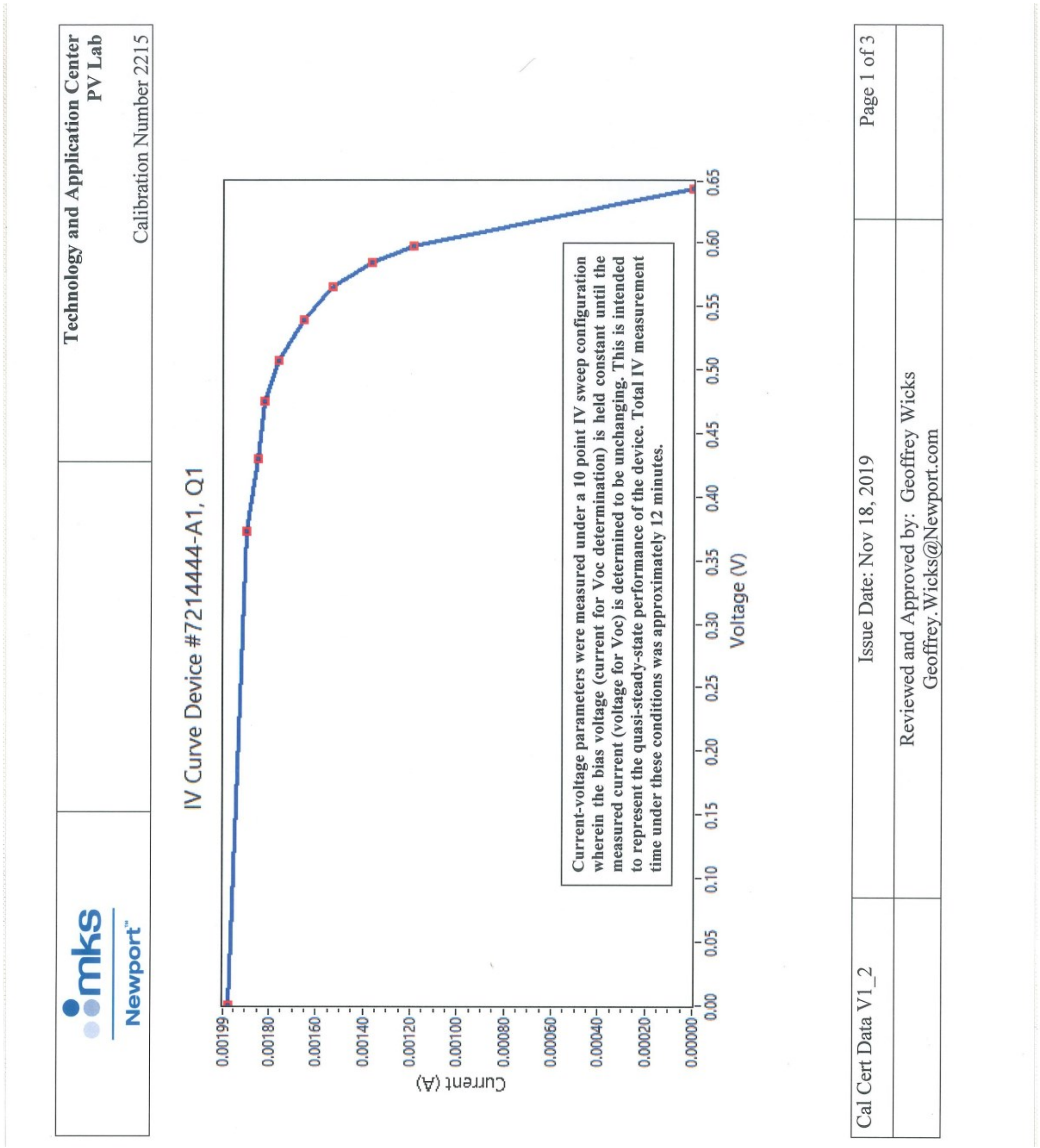

Supplementary Figure 27. Certified results of *I-V* curve

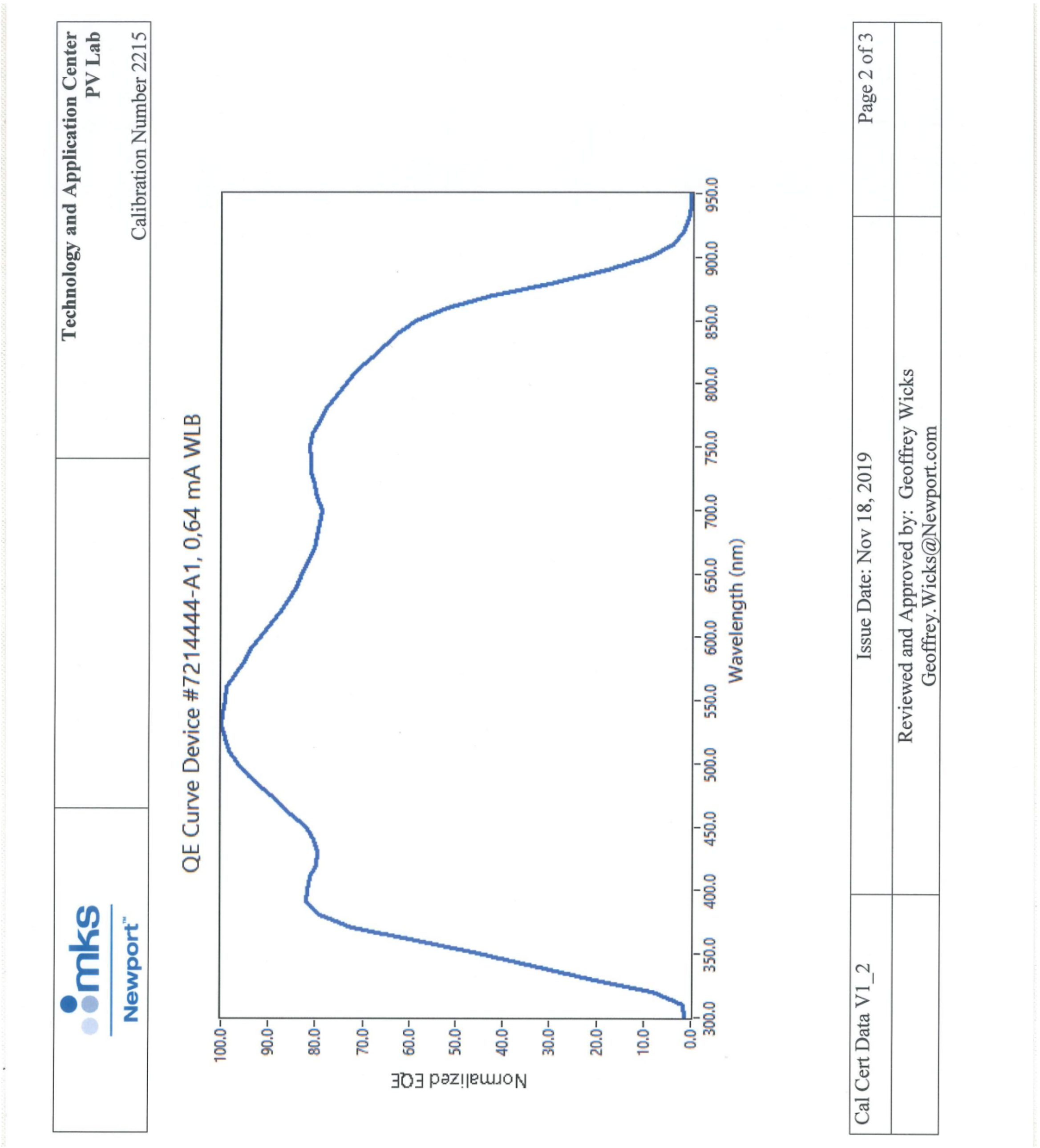

Supplementary Figure 28. Certified results of normalized external quantum efficiency

a

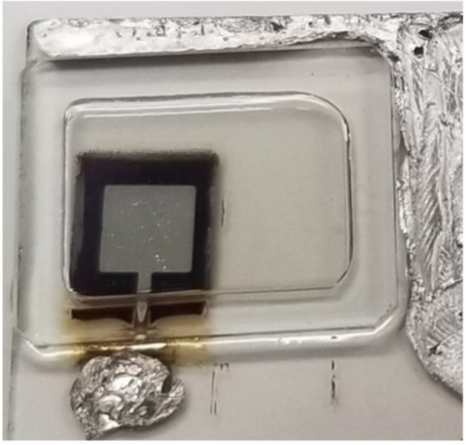

b

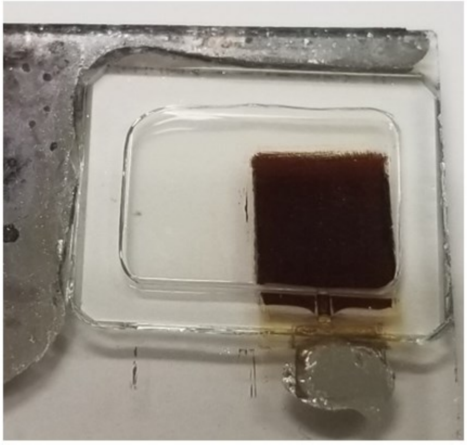

Supplementary Figure 29. The encapsulation of TPSC. (a) Front image, (b) back image.

Supplementary Table 1: Calculated surface energies for tin perovskite films with varied content of additives in precursor solutions.

Perovskite film	Surface Energy (mJ m^{-2})
Sn-1X	46.3
Sn-2X	43.8
Sn-3X	30.6
$CsFASnI_3$+10%SnF_2+10%$SnCl_2$	34.6
$CsFASnI_3$+10%SnF_2+30%$SnCl_2$	38.8
$CsFASnI_3$+20%SnF_2	42.4

Supplementary Table 2: Device parameter for typical Sn-2X and Sn-3X based TPSCs.

Perovskite	Scan direction	J_{SC} (mA cm^{-2})	V_{OC} (V)	FF (%)	PCE (%)
Sn-2X	Forward	18.63	0.55	67.4	6.91
	Reverse	18.67	0.57	68.5	7.30
Sn-3X	Forward	21.57	0.64	75.2	10.36
	Reverse	21.78	0.65	76.2	10.79
