# Supplementary material for: Efficient and stable tin perovskite solar cells enabled by amorphous-polycrystalline structure
Source: Nat Commun. 2020 May 29;11:2678. doi: 10.1038/s41467-020-16561-6 (PMC7260362; doi:10.1038/s41467-020-16561-6)
Supplement: Supplementary file 2 — Solar Cells Reporting Summary [file 41467_2020_16561_MOESM2_ESM.pdf]

nature research

Corresponding author(s): Xudong,Yang and Liyuan, Han

Solar Cells Reporting Summary

Nature Research wishes to improve the reproducibility of the work that we publish. This form is intended for publication with all accepted papers reporting the characterization of photovoltaic devices and provides structure for consistency and transparency in reporting. Some list items might not apply to an individual manuscript, but all fields must be completed for clarity.

For further information on Nature Research policies, including our data availability policy, see Authors & Referees.

Experimental design

Please check: are the following details reported in the manuscript?

1. Dimensions

Item	Yes/No	Details
Area of the tested solar cells	[X] Yes [ ] No	The area of test solar cells is 0.08920±0.0006 cm2. (Supplementary Figure 26)
Method used to determine the device area	[X] Yes [ ] No	The device area is determined by the aperture area of metal mask, which was certified by accredited institute of Newport Laboratory.

2. Current-voltage characterization

Item	Yes/No	Details
Current density-voltage (J-V) plots in both forward and backward direction	[X] Yes [ ] No	We give the J-V plots measured under both forward and backward directions in figure 3b.
Voltage scan conditions<br>*For instance: scan direction, speed, dwell times*	[X] Yes [ ] No	The J-V curves were measured under forward scan (-0.1 V to 0.7 V) or reverse scan (0.7 V to -0.1 V) by a fixed step voltage of 10 mV and delay time of 50 ms. (Characterization)
Test environment<br>*For instance: characterization temperature, in air or in glove box*	[X] Yes [ ] No	Photovoltaic properties were measured in air at room temperature near 25 celsius degree with encapsulation. (Characterization)
Protocol for preconditioning of the device before its characterization	[ ] Yes [X] No	No preconditioning was used
Stability of the J-V characteristic<br>*Verified with time evolution of the maximum power point or with the photocurrent at maximum power point; see ref. 7 for details.*	[X] Yes [ ] No	The stabilized output and maximum power point tracking were conduced. (Figure 3e, 3f)

3. Hysteresis or any other unusual behaviour

Item	Yes/No	Details
Description of the unusual behaviour observed during the characterization	[X] Yes [ ] No	We observed hysteresis that the efficiency measured at backward direction was higher than that at forward direction, which has been studied by many research groups. (Figure 3b)
Related experimental data	[X] Yes [ ] No	Figure 3b, Supplementary Table 2

4. Efficiency

Item	Yes/No	Details
External quantum efficiency (EQE) or incident photons to current efficiency (IPCE)	[X] Yes [ ] No	Figure 3c
A comparison between the integrated response under the standard reference spectrum and the response measure under the simulator	[X] Yes [ ] No	The integrated Jsc from IPCE is consistent with the Jsc from JV curve. (Figure 3b, 3c)
For tandem solar cells, the bias illumination and bias voltage used for each subcell	[ ] Yes [X] No	No tandem cells were made in this manuscript

5. Calibration

Item	Yes/No	Details
Light source and reference cell or sensor used for the characterization	[X] Yes [ ] No	Wacom Denso, WXS-155S-10, 100 mW cm-2 (Characterization)

Confirmation that the reference cell was calibrated and certified

☒ Yes ☐ No

We used a reference cell, which was calibrated by the Calibration, Standards and Measurement Team at the Research Center for Photovoltaics in AIST, Japan. (Characterization)

Calculation of spectral mismatch between the reference cell and the devices under test

☒ Yes ☐ No

The spectral mismatch is less than 3%. (Characterization)

6. Mask/aperture

Size of the mask/aperture used during testing

☒ Yes ☐ No

0.08920±0.0006 cm2, (Supplementary figure 26)

Variation of the measured short-circuit current density with the mask/aperture area

☐ Yes ☒ No

We used the same mask for all the photovoltaic measurements of devices.

7. Performance certification

Identity of the independent certification laboratory that confirmed the photovoltaic performance

☒ Yes ☐ No

Newport Laboratory, USA

A copy of any certificate(s)
Provide in Supplementary Information

☒ Yes ☐ No

Supplementary figure 26,27,28

8. Statistics

Number of solar cells tested

☒ Yes ☐ No

We have tested 16 cells for each composition under 100 mW cm-2, AM 1.5G illumination under forward bias scan. A statistical box chart was included in figure 3d.

Statistical analysis of the device performance

☒ Yes ☐ No

A statistical box chart was included in figure 3d.

9. Long-term stability analysis

Type of analysis, bias conditions and environmental conditions
For instance: illumination type, temperature, atmosphere humidity, encapsulation method, preconditioning temperature

☒ Yes ☐ No

We measured the long term stability of devices at the maximum power point under AM 1.5G light soaking with encapsulation. (Figure 3f, characterization)
